# On the earliest evolution of the mammaliaform teeth, jaw joint and middle ear

**DOI:** 10.1002/ctm2.1768

**Published:** 2024-07-19

**Authors:** Jin Meng, Fangyuan Mao

**Affiliations:** ^1^ Division of Paleontology American Museum of Natural History New York City New York USA; ^2^ Earth and Environmental Sciences Graduate Center City University of New York New York City New York USA; ^3^ Key Laboratory of Vertebrate Evolution and Human Origins Institute of Vertebrate Paleontology and Paleoanthropology Chinese Academy of Sciences Beijing China

**Keywords:** evolution, jaw joint, mammal teeth, middle ear

Mammals are characterized by having heterodont teeth, of which most of the check teeth are supported by divided roots and display diverse morphologies. With precise occlusion between upper and lower teeth, chewing function is possible and food processing is more efficient than in non‐mammalian reptiles. Mammals are also characterized by having a single‐boned lower jaw, the dentary, and thus a unique dentary‐squamosal jaw joint (DSJ) or the temporomandibular joint in human^1^ that allows transverse movement of lower jaws and teeth in mastication.^2^ Moreover, mammals have three ossicles in the middle ear: the stapes, the incus and the malleus, of which the incus and malleus are homologous to the reptilian quadrate and articular plus prearticular, respectively. In evolutionary biology, this configuration is called the definitive mammalian middle ear (DMME).^3^ These structures are interrelated in a complicated way and evolved gradually through a mosaic process during the early evolution of mammals. This process has not been clearly documented due to the rareness and fragmentary nature of fossils. In two studies published back‐to‐back in *Nature*, Mao et al.^4^, ^5^ reported two Jurassic species that are close relatives of mammals, *Feredocodon chowi* and *Dianoconodon youngi*, and present new evidence elucidating the earliest tooth diversification of Mammaliaformes (defined as the last common ancestor of Morganucodontidae and Mammalia and all its descendants^6^; Figure 1) and transformation of the reptilian jaw joint to the middle ear apparatus of mammals.

**FIGURE 1:**
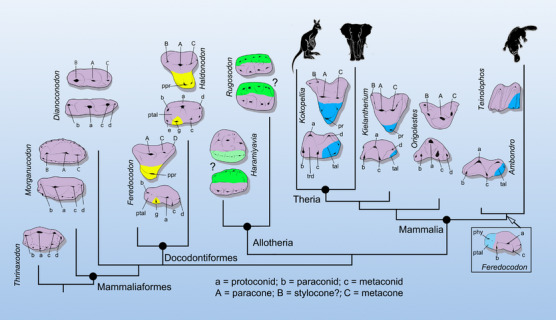
The earliest diversification of teeth in mammaliaforms. The triconodont teeth of *Morganucodon* and *Dianoconodon* represent the primitive condition that gave rise, independently, to three tooth patterns in docodontiforms, allotherians and mammals. The purple colour marks tooth parts homologous to the triconodont tooth. The yellow, green and blue colours mark neomorphic structure in the three derived groups of teeth, respectively. In conventional view, the lower molar of the shuotheriid *Feredocodon* was termed as pseudotribosphenic because its talonid is mesial to the trigonid (indicated in the boxed figure at the right lower corner), contrasting the typical tribosphenic pattern (in *Kokopellia*) where the talonid is distal to the trigonid. In addition, shuotheriids have been grouped with the clade Australosphenida containing monotremes. In Mao et al.,^4^ the cusp homology of the pseudotribosphenic pattern was reinterpreted and shuotheriids, as represented by *Feredocodon*, is clustered with docodontans to form a new clade, Docodontiformes. The figure is modified from Mao et al.^4^ phy, pseudohypoconid; ppr, pseudoprotocone; pr, protocone; ptal, pseudotalonid; tal, talonid; trd, trogonid.

## 1 EARLIEST TOOTH DIVERSIFICATIONS

Animals are what they eat. The heterodont teeth of circa 6.5 thousand extant species of mammals^7^ display remarkably diverse morphologies and efficient functions for food processing.^8^, ^9^, ^10^ These diverse teeth are presumably derived from the tribosphenic tooth pattern,^2^ as shown in opossum or the marsupial‐like fossil *Kokopellia* (Figure 1). The tribosphenic pattern may have evolved independently in the Gondwanan australosphenidans, a clade containing monotremes.^11^ In the tribosphenic teeth, the lower molar has a trigonid consisting of three triangularly arranged cusps: the protoconid, the paraconid and the metaconid. The trigonid is distally followed by a basin‐shaped talonid. The upper molar is triangular in shape, and the lingual cusp, the protocone, occludes into the talonid of the lower molar, allowing both shearing and grinding for food processing. Studying the diversity, development and evolutionary changes of mammalian dentitions is not only essential to infer the evolution of mammals but also potentially informative to understanding mechanisms of odontogenesis for clinical applications.^12^

The early evolution of mammalian teeth is not so clear as we have hoped to because of the limited fossil records. One of the existing problems concerns the pseudotribosphenic tooth pattern, first recognized in shuotheriids, an extinct group of Jurassic mammaliaforms.^13^ Reverse to the tribosphenic condition in which the talonid is distal to the trigonid, the basin‐shaped structure of the shuotheriid lower molar is mesial to the trigonid, and thus termed as pseudotalonid; accordingly, the lingual cusp of the upper molar that occludes to the pseudotalonid is the pseudoprotocone (Figure 1). The origin of the pseudotribosphenic teeth has been unclear since its proposal, obscuring our perception of shuotheriid affinities and the early evolution of mammaliaforms. The developmental study has postulated that the same developmental cascade of mammalian teeth has generated phenotypes of both the tribosphenic and pseudotribosphenic molars by emphasizing the development of the basined structure distal or mesial to the trigonid, respectively^14^ and that tooth development shows a parallel relationship with the evolution of mammalian teeth.^15^ However, extending the tooth developmental mechanism of extant mammals to the pseudotribosphenic pattern assumes that the trigonids in the tribosphenic and pseudotribosphenic patterns are homologous, which has been demonstrated to be untrue by Mao et al.^4^

The completely preserved dentitions of the Jurassic pseudotribosphenic shuotheriid, *Feredocodon chowi*, allow Mao et al.^4^ to use serial homology and occlusal relationship to reinterpret the cusp homologies of the pseudotribosphenic pattern. As a simple principle, the homologous upper and lower teeth and their cusps should have a consistent shape, relative position and occlusal relationship throughout the dentition. Mao et al. show that the previous recognition that the so‐called trigonid of the pseudotribosphenic molar is not homologous to that of the tribosphenic molar because the cusp identified as the paraconid is actually a neomorphic structure termed as cusp g, whereas the pseudohypoconid in the pseudotalonid is instead homologous to the paraconid (Figure 1). The so‐called pseudotribosphenic pattern has little to do with the tribosphenic pattern; the former is similar and homologous to the molar pattern of docodontans.

Because mammalian teeth are character rich and have played a pivotal role in reconstructing mammal phylogenies, the reinterpreted pseudotribosphenic tooth pattern triggered a series of changes of many dental characters and character coding used in phylogenetic reconstruction, which results in a new phylogeny of mammaliaforms. Previously, the pseudotribosphenic shuotheriids have been clustered with australosphenidans (Figure 1). This relationship has remained contentious because it is difficult to explain why the pseudotalonid shuotheriids forms the outgroup of the tribosphenic australosphenidans. The phylogenetic analysis based on new evidence removed shuotheriids from australosphenidans and cluster them with docodontans to form a new clade, Docodontiformes, which share the pseudotribosphenic features (Figure 1).

The new phylogeny predicts that mammaliaforms may have been derived from advanced non‐mammaliamorph cynodonts, such as *Thrinaxodon*, because their teeth are highly similar^4^ (Figure 1). The tooth pattern ancestral to mammaliaforms, typically present in morganucodontans, is triconodont that has three main cusps aligned in a row and supported by divided roots. This triconodont tooth pattern gave rise to three major tooth patterns independently in allotherians, docodontiforms and the lineage leading to mammals. The common modification of the tooth patterns in the three groups of mammaliaforms is the transverse (labio‐lingual) widening of the posterior teeth, particularly the molars. Tooth widening in allotherians is probably achieved by the addition of an extra cusp row^4^ (Figure 1), but convincing evidence for this process is needed. Tooth widening in docodontiforms was achieved in the upper molars by the development of a neomorphic pseudoprotocone and in the lower molars by the addition of cusp g and semitriangulation of the main cusps in which cusp c rotates lingually in relation to cusp a (Figure 1). Deviation of the symmetrodont and tribosphenic molar patterns from that of morganucodontans has been well established.^4^ Tooth widening in this lineage was achieved by cusp triangulation (both cusp b and c rotating lingually to cusp a) and by the development of the talonid in the lower molars and by the development of the neomorphic protocone in the upper molar at late stages of evolution. The study supports the view that the processes involved in the evolution of the pseudotribosphenic and tribosphenic molars are fundamentally different.^16^ This is consistent with the jaw and middle ear structures: The pseudotribosphenic molars are associated with a primitive jaw in which the postdentary bones (middle ear bones) are still attached to the dentary, whereas tribosphenic mammals have the postdentary bones detached, as we will discuss below. The tooth developmental mechanism of extant mammals may explain fossil tooth pattern related to the tribosphenic teeth,^14^, ^15^ but may not replay the dental evolutionary transition of the extinct groups. The developmental mechanism of the pseudotribosphenic teeth, probably the allotherian teeth as well, was likely lost during the evolution of mammals.^4^ Nonetheless, these diverse dental patterns showed ecomorphological specializations and diversification during the earliest stage of mammaliaform evolution.

## 2 EVOLUTION OF JAW AND MIDDLE EAR

**FIGURE 2:**
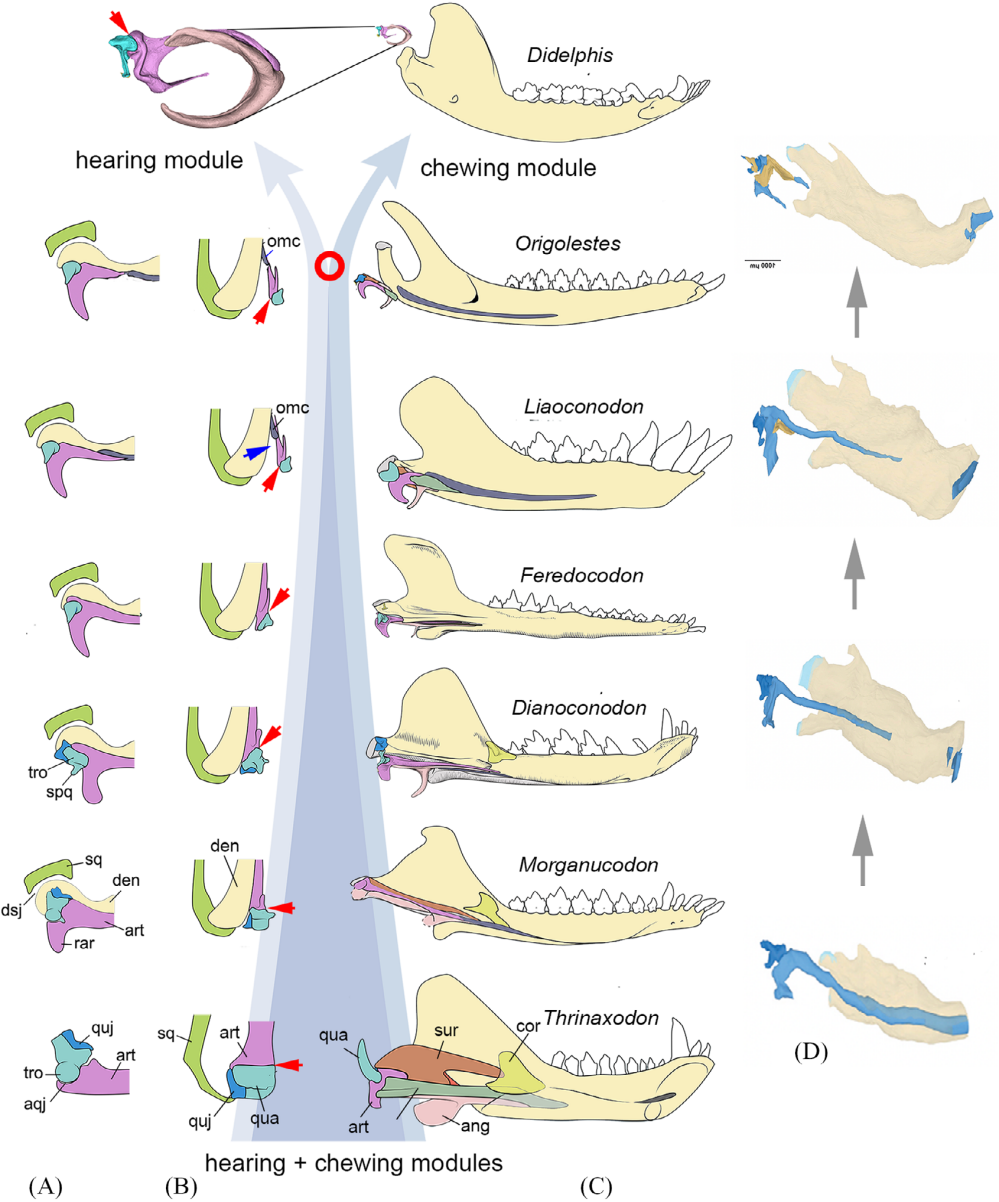
Diagram outlining the evolution of the mammalian middle ears in comparison with the development sequence. (A, B) The primary jaw joint (PJ) between the articular (pink)‐quadrate (light blue) in lingual and ventral views and its transformation to the middle ear ossicles of mammals. For simplicity, only the articular bone of the post‐dentary unit is shown in the sketches. The red arrow points to the PJ and the blue arrow indicates detachment of the postdentary bones from the dentary. (C) Lingual views of the lower jaws from representative mammals and kin (see also Figure 1), showing the primary joint and postdentary bones in relation to the dentary bones (A−C modified from Mao et al.^5^, ^22^). (D) Various developmental stages of the lower jaw and middle ear bones in mouse, showing the change of the dual jaw joints, roughly recapitulating the evolutionary process (modified from Fernández‐Rubio and Radlanski^19^). The background arrows indicate the trend from the intermixed hearing and chew modules in the close kin of mammals to the decoupling of the two systems in mammals (marked by the red circle). Figures are not on the scale. ang, angular; aqj, articular‐quadrate joint (primary joint); art, articular; cor, coracoid; den, dentary; dsj, dentary‐squamosal joint (secondary joint); omc, ossified Meckel's cartilage; qua, quadrate; quj, quadratojugual; sq, squamosal; spq, stapedial process of the quadrate; sur, surangular; tro, trochlea of the quadrate.

In adult mammals, the middle ear ossicles and DSJ are fully separated systems that function for hearing and chewing, respectively. The DSJ as a unique joint in mammals forms via a distinct molecular programme^17^ and allows transverse jaw movement during mastication.^2^ However, the two systems and their functions are intermixed in close relatives of mammals in the Mesozoic. In mammal‐like reptiles, the jaw joint is exclusively between the articular bone in the mandible and the quadrate bone in the cranium. At an evolutionary stage represented by a Late Triassic‐Early Jurassic (about 200 million years ago) mammaliaform, *Morganucodon*, the DSJ was developed as a secondary joint lateral to the articular‐quadrate joint, also termed the primary joint (PJ) so that *Morganucodon* possesses a dual jaw joint^18^ (Figure 2). In the dual jaw joint, the DSJ operates as the main load‐bearing articulation for the jaw function, whereas the PJ and associated postdentary bones (the articular, prearticular, angular and surangular) reduced their size and role played in the jaw joint while shifting towards hearing function; such a hearing apparatus is called the mandibular middle ear (MdME).^5^ The similar dual joint and function exist in the early stage of development in extant mammals. In placentals, including human, the dual joint occurs in the prenatal stage^19^ (Figure 2). In marsupials and monotremes, the dual joints, or similar structure, occur in the postnatal stage and the middle ear functions as part of the mandible until the DSJ forms.^20^

The process in that the load‐bearing PJ lost its original function and became part of the auditory apparatus has remained unclear. Mao et al.^5^ show that the morganucodontan‐like *Dianoconodon* differs from *Morganucodon* in having a more derived MdME in which the postdentary bones, the quadrate and quadratojugal are further reduced in size, the stapes has slim crura and the quadrate is more medially shifted with its trochlea orientated diagonally in relation to the articular; these features indicate that the PJ of *Dianoconodon* could not bear much stress during mastication. The MdME of *Feredocodon* is further derived than that of *Dianoconodon*. It has slimmer and shorter post‐dentary bones and the quadrate approaches the mammalian condition in lacking the trochlea and compressed (shortened); it is further medially shifted in relation to the articular. This, along with the loss of the quadratojugal, allows more flexibility of the quadrate and indicates that the PJ has completely lost its load‐bearing function and works exclusively for hearing, although the hearing function would be interfered by chewing because these auditory bones are still lodged in the post‐dentary trough.

Mao et al.’s work identified a key change in the process of freeing the PJ from load‐bearing jaw function: It is the medial shift of the reduced quadrate in relation to the articular, which gradually optimizes the quadrate as an element transmitting airborne sound vibrations from the tympanic membrane to the stapes.^5^ Interrelated with the quadrate shift, other features in this system transformation involved the shortening of the post‐dentary bones and the reduction − and eventually the loss − of the quadratojugal. Furthermore, the slimmed stapes is no longer structurally suitable for resisting the medial displacement of the quadrate during the opening and closing of the jaw, but becomes more efficient for sensing airborne sounds. These changes are echoed by the well‐developed reflected lamina of the angular bone and the retroarticular process of the articular bone, presumably for attachment of the tympanic membrane. The release of the PJ from the jaw articulation is a precondition for the detachment of the post‐dentary bones from the dentary, which leads to the transitional mammalian middle ear (TMME), best represented by the Early Cretaceous euthriconodon *Liaoconodon* ^21^ (Figure 2). In the TMME, the postdentary bones have detached from the dentary and functioned as the auditory apparatus, but the anterior process of the malleus is still attached to the posterior end of the ossified Meckel's cartilage (OMC); the latter is anteriorly lodged in the Meckelian groove of the dentary bone; thus, the chewing and hearing functions are still linked by the OMC in the TMME. The middle ear of the Early Cretaceous symmetrodont mammal, *Origolestes*, displays a snap‐shot, documenting a more derived evolutionary stage when the middle ear bones were eventually separated from the OMC so that the hearing and chewing systems and functions are finally decoupled^22^ (Figure 2). Without interfering with each other, the decoupled hearing and chewing apparatuses could evolve more efficiently for their own function, respectively, and, with greater evolvability, resulted in diverse morphologies in both the jaw joint and the middle ear ossicles in extant mammals.

The new fossils show that the OMC had a pivotal role during the migration of the post‐dentary bones from the dentary to the basicranium. In *Dianoconodon* and *Feredocodon*, the OMC is a slim rod located anteriorly to the post‐dentary bones; it becomes a robust element and migrates posteriorly in the dentary of some Mesozoic mammals (Figure 2) and probably provides a stabilizing mechanism for the detached ossicles that have not been moored in the basicranial region^21^ (Figure 2). However, because of the sparse fossil records, some critical stages, such as the formation of the dentary‐squamosal joint during the evolution, have only been poorly known. On the other hand, loss of the quadratojugal and surangular bones during the evolution is not reflected in the development of extant mammals. Much still remains to be learned. Nonetheless, the available fossil evidence strongly supports the hypothesis of a gradual evolution from the dual jaw joint in *Morganucodon* to the DMME in mammals and this process is partly recapitulated in the development of extant mammals.^19^, ^20^

## AUTHOR CONTRIBUTIONS

The commentary is written by Jin Meng and Fangyuan Mao based on the studies published in *Nature* (Mao et al.^4^, ^5^).

## CONFLICT OF INTEREST STATEMENT

We have no competing interests to declare.

## ETHICS STATEMENT

This study was based on fossil specimens; no living animals were involved. The fossils were collected under the ethics oversight of the Institute of Vertebrate Paleontology and Paleoanthropology, Chinese Academy of Sciences, Beijing and the American Museum of Natural History, New York, New York, USA.

## References

[1] Mansour S , Magnan J , Ahmad HH , Nicolas K , Louryan S . Comprehensive and Clinical Anatomy of the Middle Ear. Springer‐Verlag Berlin; 2019.

[2] Crompton AW , Hiiemae K . Molar occlusion and mandibular movements during occlusion in the American opossum, *Didelphis marsupialis* . Zool J Linn Soc. 1969;49(1):21‐47.

[3] Allin EF , Hopson JA . The Evolutionary Biology of Hearing. New York: Springer New York; 1992.

[4] Mao F , Li Z , Wang Z , et al. Jurassic shu otheriids show earliest dental diversification of mammaliaforms. Nature. 2024;628(8008):569‐575. 38570681 10.1038/s41586-024-07258-7

[5] Mao F , Zhang C , Ren J , et al. Fossils document evolutionary changes of jaw joint to mammalian middle ear. Nature. 2024;628(8008):576‐581. 38570677 10.1038/s41586-024-07235-0

[6] Rowe T . Definition, diagnosis, and origin of mammalia. J Vertebr Paleontol. 1988;8:241‐264.

[7] Burgin CJ , Colella JP , Kahn PL , Upham NS . How many species of mammals are there? J Mammal. 2018;99(1):1‐14.

[8] Lucas PW . Dental Functional Morphology: How Teeth Work. Cambridge University Press; 2004.

[9] Hillson S . Teeth. Cambridge University Press; 2005.

[10] Ungar PS . Mammal Teeth: Origin, Evolution and Diversity. Hopkins University Press; 2010.

[11] Luo ZX , Cifelli RL , Kielan‐Jaworowska Z . Dual origin of tribosphenic mammals. Nature. 2001;409(6816):53‐57. 11343108 10.1038/35051023

[12] Popowics T , Mulimani P . Mammalian dental diversity: an evolutionary template for regenerative dentistry. Front Dent Med. 2023;4:1158482. 10.3389/fdmed.2023.1158482 PMC11797774 39916902

[13] Chow MC , Rich TH . *Shuotherium dongi*, n. gen. and sp., a therian with pseudo–tribosphenic molars from the Jurassic of Sichuan, China. Aust Mammal. 1982;52:127‐142.

[14] Harjunmaa E , Seidel K , Häkkinen T , et al. Replaying evolutionary transitions from the dental fossil record. Nature. 2014;512:44‐48. 25079326 10.1038/nature13613 PMC4252015

[15] Yamanaka A . Evolution and development of the mammalian multicuspid teeth. J Oral Biosci. 2022;64(2):165‐175. 35390544 10.1016/j.job.2022.03.007

[16] Crompton AW , Jenkins FA . Molar occlusion in Late Triassic mammals. Biol Rev. 1968;43:427‐458. 4886687 10.1111/j.1469-185x.1968.tb00966.x

[17] Purcell P , Joo BW , Hu JK , et al. Temporomandibular joint formation requires two distinct hedgehog‐dependent steps. Proc Natl Acad Sci USA. 2009;106(43):18297‐18302. 19815519 10.1073/pnas.0908836106 PMC2775291

[18] Kermack KA , Mussett F , Rigney HW . The skull of *Morganucodon* . Zool J Linn Soc. 1981;71(1):1‐158.

[19] Fernández‐Rubio EM , Radlanski RJ . Development of the primary and secondary jaw joints in the mouse. Ann Anat. 2023;249:152085. 36940887 10.1016/j.aanat.2023.152085

[20] Anthwal N , Fenelon JC , Johnston SD , Renfree MB , Tucker AS . Transient role of the middle ear as a lower jaw support across mammals. eLife. 2020;9:e57860. 32600529 10.7554/eLife.57860 PMC7363448

[21] Meng J , Wang Y , Li C . Transitional mammalian middle ear from a new Cretaceous Jehol eutriconodont. Nature. 2011;472(7342):181‐185. 21490668 10.1038/nature09921

[22] Mao F , Hu Y , Li C , et al. Integrated hearing and chewing modules decoupled in a Cretaceous stem therian mammal. Science. 2020;367(6475):305‐308. 31806694 10.1126/science.aay9220

